# DDRugging glioblastoma: understanding and targeting the DNA damage response to improve future therapies

**DOI:** 10.1002/1878-0261.13020

**Published:** 2021-06-11

**Authors:** Ola Rominiyi, Spencer J. Collis

**Affiliations:** ^1^ Weston Park Cancer Centre Sheffield UK; ^2^ Department of Oncology & Metabolism The University of Sheffield Medical School UK; ^3^ Department of Neurosurgery Royal Hallamshire Hospital Sheffield Teaching Hospitals NHS Foundation Trust UK; ^4^ Sheffield Institute for Nucleic Acids (SInFoNiA) University of Sheffield UK

**Keywords:** chemotherapy, DNA damage response, glioblastoma, radiotherapy, synthetic lethality, tumour‐treating fields

## Abstract

Glioblastoma is the most frequently diagnosed type of primary brain tumour in adults. These aggressive tumours are characterised by inherent treatment resistance and disease progression, contributing to ~ 190 000 brain tumour‐related deaths globally each year. Current therapeutic interventions consist of surgical resection followed by radiotherapy and temozolomide chemotherapy, but average survival is typically around 1 year, with < 10% of patients surviving more than 5 years. Recently, a fourth treatment modality of intermediate‐frequency low‐intensity electric fields [called tumour‐treating fields (TTFields)] was clinically approved for glioblastoma in some countries after it was found to increase median overall survival rates by ~ 5 months in a phase III randomised clinical trial. However, beyond these treatments, attempts to establish more effective therapies have yielded little improvement in survival for patients over the last 50 years. This is in contrast to many other types of cancer and highlights glioblastoma as a recognised tumour of unmet clinical need. Previous work has revealed that glioblastomas contain stem cell‐like subpopulations that exhibit heightened expression of DNA damage response (DDR) factors, contributing to therapy resistance and disease relapse. Given that radiotherapy, chemotherapy and TTFields‐based therapies all impact DDR mechanisms, this Review will focus on our current knowledge of the role of the DDR in glioblastoma biology and treatment. We also discuss the potential of effective multimodal targeting of the DDR combined with standard‐of‐care therapies, as well as emerging therapeutic targets, in providing much‐needed improvements in survival rates for patients.

## Graphical abstract

Glioblastoma remains a cancer of unmet clinical need. Current treatment incorporates DNA damaging therapies which activate an intricate, multifaceted DNA damage response (DDR). This Review discusses the role of the DDR in the complex biology and therapeutic response of glioblastoma. Additionally, novel multimodal approaches to target the DDR alongside standard‐of‐care therapies are presented, towards the goal of improving patient survival.

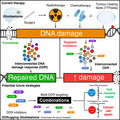

**Abbreviations**
ATM: ataxia telangiectasia mutated
ATR: ataxia telangiectasia and Rad3‐related kinase
BBB: blood–brain barrier
BER: base excision repair
BRCA: breast cancer gene
CDK: cyclin‐dependent kinase
cGAS: cyclic GMP‐AMP synthase
CSCs: cancer stem cells
DDR: DNA damage response
DNA‐PK: DNA‐dependent protein kinase
DSB: double‐strand break
ERK5: extracellular‐related signalling kinase 5
FA: Fanconi anaemia
FDA: Food and Drug Administration (USA)
GSCs: glioma stem‐like cells
HGG: high‐grade glioma
HR: homologous recombination
ICL: interstrand crosslink
IDH: isocitrate dehydrogenase
IR: ionising radiation
MGMT: methylguanine methyltransferase
MMR: mismatch repair
MRI: magnetic resonance imaging
NER: nucleotide excision repair
NHEJ: nonhomologous end joining
O6MeG: O6‐methylguanine
PARP: poly (ADP‐ribose) polymerase
PIKK: phosphatidylinositol 3‐kinase‐related kinases
ROS: reactive oxygen species
RSS: replication stress signalling
SSB: single‐strand break
SSL: synthetic sensitivity or lethality
STING: stimulator of interferon genes
TMZ: temozolomide
TTFields: tumour‐treating fields
WHO: World Health Organization

## 1 Introduction

### 1.1 Glioblastoma and current treatment regimes

Brain tumours are globally responsible for around 190 000 deaths per year (around 5000 of which are in the UK) and are responsible for the greatest reduction in life expectancy of any cancer – around 20 years on average [1, 2]. Glioblastoma is ascribed the highest glioma grade designated by the World Health Organization (WHO grade IV glioma). It is the most frequently diagnosed primary brain tumour in adults and is associated with an exceptionally poor clinical course characterised by treatment resistance, rapid disease progression and dire patient survival rates of around 12–15 months following diagnosis in clinical studies [3]. However, average survival for unselected patients in the real‐world setting is typically closer to 8 months [4]. In most cases, glioblastoma arises rapidly without previous clinical presentation or histological confirmation of a less malignant precursor lesion, although, in a minority of cases, signs of progression from a lower grade diffuse (WHO grade II) or anaplastic (WHO grade III) astrocytoma are evident [5].

Despite some recent advances in the genetic, epigenetic and molecular subtyping of gliomas [6], the current standard‐of‐care treatment remains maximal safe surgical resection followed by a course of radiotherapy with concomitant and adjuvant chemotherapy [7, 8, 9]. The mainstay chemotherapeutic agent is oral delivery of the DNA alkylating agent temozolomide following successful clinical trial data evidencing a 2.5‐month increase in patient survival rates [7, 8]. However, despite this aggressive course of therapy, median patient survival was estimated at 14.6 months, with < 10% of patients surviving more than 5 years. Importantly, however, in the subset of patients with promoter methylation of the DNA repair gene *MGMT* (discussed below), the addition of temozolomide was associated with overall survival of 21.7 months, representing an increase of 6.4 months [10]. More recent studies corroborate the benefit of temozolomide in the context of methylated *MGMT* status [11, 12]. However, in contrast, efforts to improve the survival of patients with an unmethylated *MGMT* promoter have been less successful [13, 14].

Since the approval of temozolomide in 2005, no new approved treatments for glioblastoma were forthcoming until the recent development and clinical approval of tumour‐treating fields (TTFields) therapy, which uses locoregional delivery of alternating electrical fields within a narrow frequency range to specifically target rapidly dividing cancer cells within a defined brain region [15, 16, 17]. Akin to the introduction of temozolomide into existing radiotherapy regimes, the inclusion of TTFields to current temozolomide dosing schedules increased overall patient survival rates by approximately 5 months, leading to clinical approval for newly diagnosed glioblastoma by the Food and Drug Administration (FDA) in the United States [17] in 2015, following its initial approval for recurrent glioblastoma in 2011. However, the incorporation of TTFields into standard‐of‐care therapy for glioblastoma is not universally accepted for numerous reasons, including the lack of a placebo treatment, such as a sham TTFields device, in key phase 3 randomised trials [17, 18]; incomplete understanding of the mechanistic basis for TTFields therapeutic effects [16]; the challenge of maintaining high treatment compliance; and the current high financial costs associated with this treatment regime. Consequently, opportunities for patients to receive TTFields are not geographically equitable and are largely dependent on the country’s healthcare system approval structure for cancer therapies [16]. Importantly, and relevant to this Review, the current landscape of multimodal therapy used to treat glioblastoma – that is, chemotherapy, radiotherapy and TTFields – all induces DNA damage, replication stress and mitotic‐mediated cell death within tumour cells. As such, a deeper understanding of the innate DNA damage response (DDR) mechanisms and coordinated cell cycle regulation within these tumours offers opportunities to uncover exploitable tumour‐specific genetic and phenotypic vulnerabilities which could lead to more efficient targeting and effective treatment strategies in the clinic to confer much‐needed improvements in survival rates for patients.

### 1.2 Therapeutic challenges for glioblastoma

A cornerstone of glioblastoma treatment is effective surgical resection of the tumour bulk. However, clinical studies have suggested that neurosurgical removal of as much as 98% of the tumour volume may be required to provide an impact on median survival [19]. As such, a plethora of surgical innovations have been applied to help maximise surgical resection rates. Multimodal neuronavigation, intraoperative magnetic resonance imaging and ultrasound (MRI/US), and fluorescence‐guided surgery with 5‐aminolevulinic acid (5‐ALA) have demonstrated the potential to augment surgical reduction of tumour burden [20, 21, 22, 23, 24]. Additionally, forthcoming innovations, including the application of iKnife, which utilises rapid evaporative ionisation mass spectrometry (REIMS) to differentiate between tumour and healthy tissue [25, 26] or Raman spectroscopy, which is used to achieve similar tumour‐brain resolution in a nondestructive manner [27, 28, 29, 30], may yield further improvements in resection rates in the near future. However, given the highly infiltrative nature of glioblastoma and limited ability to resect infiltrated regions of eloquent cortex without unacceptable morbidity, it is unfortunately hard to envisage advances in surgical technology impacting patient survival outcomes beyond incremental gains.

In a similar manner to surgical interventions, radiotherapy has also seen technological advancements, including intensity‐modulated radiation therapy, stereotactic radiosurgery and proton beam therapy, which have the potential to lead to improved delivery of effective radiation doses to tumour sites whilst sparing the surrounding healthy brain tissue [31, 32].

Beyond the challenges associated with effective and maximal surgical resection and targeted radiotherapy regimes, delivery of a therapeutically effective dose of chemotherapy to postsurgical residual tumour cells is hampered by the presence of the blood–brain barrier/blood–tumour barrier (BBB) [3, 33], although there are several groups developing ways in which to disrupt or circumvent the BBB (discussed later).

The development of effective future therapies for glioblastoma will also need to tackle the biological mechanisms that help facilitate treatment resistance in these aggressive cancers. Unlike lower grade tumours, the majority of glioblastomas are classed as isocitrate dehydrogenase (IDH) wild‐type but often exhibit mutations and/or deletions in phosphatase and tensin homolog (*PTEN*), cyclin‐dependent kinase inhibitor 2A/B (*CDKN2A/B*), telomerase reverse transcriptase (*TERT*) promoter, tumour protein P53 (*TP53*), neurofibromin 1 (*NF1*), phosphatidylinositol‐4,5‐bisphosphate 3‐kinase catalytic subunit alpha (*PIK3CA*) and phosphoinositide‐3‐kinase regulatory subunit 1 (*PIK3R1*), as well as amplification of epidermal growth factor receptor (*EGFR*) and/or gain of chromosome 7, chromosome 10 monosomy and *MGMT* promoter methylation [5, 34, 35, 36]. However, at present, no targeted therapy designed to exploit such genetic and/or phenotypic traits has proven successful within the clinic [37]. One of the main reasons for this is that glioblastomas exhibit profound intratumoural heterogeneity, with spatiotemporally divergent subpopulations within a tumour displaying varying profiles of vulnerability and resistance [38, 39, 40, 41], which likely contributes to local disease recurrence. Additionally, increasing evidence supports the existence of a subpopulation of glioblastoma cells that possess an innate capacity for unlimited regeneration and self‐renewal, which are often described as cancer stem cells (CSCs) or glioblastoma stem cells (GSCs) [3, 42, 43, 44, 45, 46, 47]. Importantly, GSCs are often deemed responsible for resistance to conventional chemoradiotherapy treatment regimens through enhanced activation of DNA damage checkpoints and DNA repair capacity [48, 49, 50]. This will be highlighted with specific examples later within this Review.

### 1.3 Clinically relevant models of glioblastoma

Over the last decade, there has been a disappointing lack of success in translating promising novel agents or drug repurposing from preclinical studies into clinical survival benefit for patients with glioblastoma [51]. To some degree, this failure is representative of a lack of clinically and postsurgically relevant preclinical models of glioblastoma. In particular, the reported and further emerging intratumoural heterogeneity that exists within gliomas is a vital consideration for the development of future therapies. Variation in the genetic aberrations, phenotypic characteristics and clinical progression of individual patient tumours presupposes the need for personalised therapeutic strategies informed by a range of molecular biomarkers [52, 53, 54, 55, 56]. Furthermore, accumulating evidence suggests that a phylogenetic hierarchy of spatiotemporally divergent subclones exists within individual glioblastomas, each possessing a distinct collection of putative driver mutations and a characteristic transcriptome [38, 40, 53, 57, 58]. Creating appropriate preclinical models that are capable of recapitulating such profound intratumoural heterogeneity as well as GSC states represents a key challenge for the development of new therapeutic agents that can impact patient survival [3, 46, 59, 60]. A detailed discussion of the advantages and disadvantages of the various models is beyond the scope of this Review, but these currently consist of *in vitro* preclinical glioblastoma models that range from traditional commercially available immortalised cell lines or fresh patient‐derived primary cell cultures (‘bulk’ or stem cell enriched) which can all be maintained in either two‐ or three‐dimensional (2D or 3D) architectures, as well as more complex tumouroid and organoid cocultures and microfluidic ‘glioblastoma‐on‐a‐chip’ cultures [61, 62, 63, 64, 65, 66, 67, 68, 69, 70, 71, 72, 73]. Likewise, there are a plethora of *in vivo* preclinical glioblastoma models available, including subcutaneous, syngeneic, orthotopic and patient‐derived (PDX) xenografts as well as *de novo* genetically engineered rodent models [74, 75, 76, 77].

What is clear from the historic lack of newly approved therapies and limited improvement in patient survival rates over the last 50 years compared with other solid tumours is that glioblastoma represents a complex and difficult therapeutic challenge. However, recent work by numerous groups has started to reveal further genetic and molecular insights into glioblastoma biology, particularly within the area of DDR mechanisms that are triggered by current standard‐of‐care radiochemotherapy regimes used to treat these tumours. Furthermore, an evolving understanding of the GSC niche together with the development of novel preclinical models that better reflect postsurgical residual disease should hopefully begin to yield new therapeutic strategies over the next 5–10 years.

## 2 The DNA damage response and glioblastoma treatment

### 2.1 Cellular responses to DNA damage

The structural integrity of DNA is constantly threatened due to replication stress, telomere attrition and a multitude of endogenous and exogenous agents that generate high levels of varying DNA lesions. Such agents include metabolic by‐products, such as reactive oxygen species (ROS) and aldehydes, UV light, ionising radiation (IR) and chemical toxins [78, 79, 80]. Failure to repair DNA lesions induced by these processes can lead to mutagenesis, tumorigenesis or cell attrition. Given the potential deleterious effects on genome integrity induced by such lesions, it is perhaps not surprising that an intricate network of signalling pathways and reparative mechanisms have evolved to deal with a plethora of DNA lesions, preserving genomic architecture and integrity (genome stability). This network is collectively referred to as the DNA damage response (DDR), which encompasses a coordinated and interconnected network of pathways that regulate cell cycle progression/checkpoints, DNA repair mechanisms, DNA replication and mitotic progression, as well as transcriptional and cell death processes, in order to preserve the integrity of the genome [79, 81, 82, 83, 84, 85]. Importantly, given that radiation and chemotherapy treatments cause DNA lesions and affect cell cycle progression, heightened and/or dysregulated DDR mechanisms within tumour cells can often give rise to innate and/or acquired treatment resistance. However, such dysregulation to DDR mechanisms/processes during cancer development and progression can also offer vital cancer‐selective vulnerabilities that can be exploited for an improved therapeutic index as part of either monotherapy‐ or combination therapy‐targeted treatment strategies [82, 85, 86, 87, 88, 89].

The mechanisms associated with physically repairing DNA damage can be categorised into four main types based on the type of DNA lesion present: (a) base modifications (including alkylation damage) and mispaired bases; (b) intrastrand or interstrand DNA crosslinks; (c) single‐strand breaks (SSBs); and (d) double‐strand breaks (DSBs) [79, 81, 84, 85, 90]. Additionally, although DSBs are often considered amongst the most toxic of DNA lesions, it is worth considering that each type of lesion is not an enduringly separate entity. For example, radiation‐induced damage often leads to so‐called ‘complex lesions’ or ‘clustered damage’, in which multiple lesions are present within a few hundred base pairs of the DNA helix [91]. The collision of replication forks with SSBs or DNA interstrand crosslinks (ICLs), particularly as a consequence of oncogene activation, can result in the formation of DSBs and other deleterious lesions. Similarly, DNA : RNA hybrids known as R‐loops can be generated from replication forks colliding with transcription bubbles, which can have a plethora of deleterious effects if not adequately resolved [92, 93]. As such, the dynamic relationships that exist between various DNA lesions lead to functional redundancy within the reparative processes, which likely contributes to treatment resistance and forms the rationale for targeting multiple DDR pathways/processes to potentially overcome treatment resistance [78, 82, 83, 86, 87, 88, 89].

**Fig. 1:**
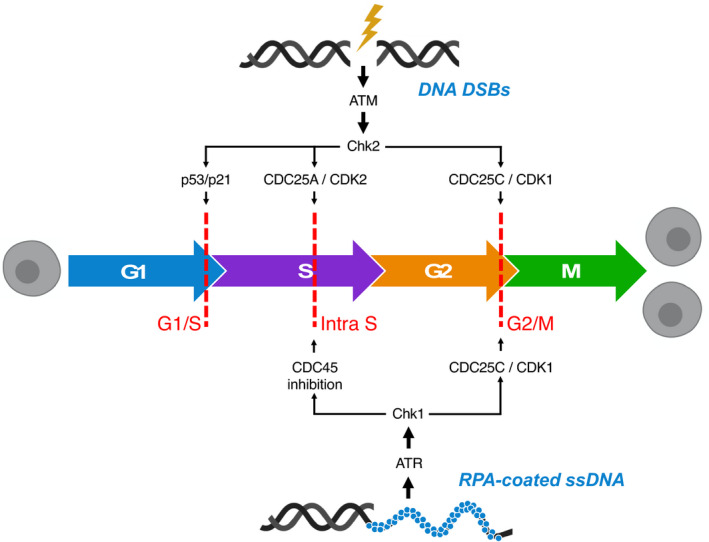
The role of ATM and ATR in cell cycle regulation following DNA damage. The processes of cell division (mitosis, M phase) and DNA synthesis (S phase) are separated by two important gap phases (G1 and G2). Progression of mitotic cells through the cell cycle is controlled by periodic accumulation and destruction of the aptly named cyclin‐dependent kinases (CDKs) and cyclins. Inappropriate progression through phases of the cell cycle is prevented by three main checkpoints (G1/S, intra‐S and G2/M checkpoints; dashed red lines). Following DNA damage, checkpoint activation is critical to provide ample time and recruit the necessary machinery required to maintain genomic integrity. *Checkpoint activation*: DNA double‐strand breaks (DSBs) activate the apical DNA damage response (DDR) kinase ataxia telangiectasia mutated (ATM), which can influence all three major cell cycle checkpoints via the phosphorylation of checkpoint kinase 2 (CHK2) and subsequent downstream signalling. In contrast, ataxia telangiectasia and Rad3‐related kinase (ATR) is activated by the presence of replication protein A (RPA)‐coated single‐stranded DNA (ssDNA) and contributes to maintenance of the intra‐S phase and G2/M checkpoints via phosphorylation of checkpoint kinase 1 (CHK1) and subsequent downstream signalling as indicated. *G1/S checkpoint*: Phosphorylation of p53 by CHK2 and ATM directly (arrow not shown) results in a reduction in the binding of mouse double minute 2 homolog (MDM2) to p53 and p53 activation, promoting its nuclear accumulation and stabilisation. Subsequently, elevated p53 levels promote increased transcription of p21, which inhibits CDK2–cyclin‐E activity, resulting in prevention of progression to S phase. *Intra‐S checkpoint*: Within S phase, the activation of cell division cycle 25 (CDC25) phosphatases predominantly by prevention of cell division cycle 45 (CDC45) loading onto replication origins (preventing subsequent DNA replication) primarily via the ATR–CHK1 axis, but also via ATM‐CHK2‐mediated phosphorylation of CDC25A, can instigate an intra‐S checkpoint in response to replication stress or other perturbations to optimal DNA synthesis, permitting a slowing of DNA replication. *G2/M checkpoint*: Both ATM‐ and ATR‐mediated phosphorylation of CHK2 and CHK1, respectively, lead to the phosphorylation of CDC25C phosphatases, which influence the G2/M checkpoint via interaction with the cyclinB1–CDK1 complex. This figure is adapted, with permission, from Ref. [227].

Maintenance of genome integrity relies not only on accurate DNA repair, but also on the processes that detect DNA damage and co‐ordinate cell cycle checkpoints, allowing time for DNA to be repaired. The regulation of cell cycle checkpoints and subsequent DNA repair processes as part of the early DDR process is controlled by three main related protein kinases – ataxia telangiectasia mutated (ATM); ataxia telangiectasia and Rad3‐related kinase (ATR); and DNA‐dependent protein kinase (DNA‐PK) – along with the various cyclin‐dependent kinases (CDKs) and the central cell cycle regulator p53 and its associated factors [83, 90] (Fig. 1). ATM represents an apical, multifunctional kinase within the DDR and typically plays a key role in cellular responses to DNA DSBs, where it helps co‐ordinate all major cell cycle checkpoints via the ATM‐CHK2 axis [90, 94]. Through the ATR‐CHK1 signalling axis, ATR helps preserve DNA integrity in response to replication stress (perturbation to ongoing replication forks) [95], although it is important to note that there is significant crosstalk and some functional redundancy amongst the activities of these related kinases [90, 96]. However, due to the main differing functions of these kinases, they are considered as credible separate drug targets in cancer biology as part of either monotherapy or combinatorial (adjuvant) therapeutic approaches [88, 90, 97, 98].

Beyond the initial DDR signalling and cell cycle checkpoint regulation, a key part of the cellular responses to DNA damage is obviously the physical repair of the vast array of DNA lesions that can be present within both heterochromatic and euchromatic regions of the genome [99, 100, 101, 102]. An exhaustive discussion of the vast array of DDR pathways is not possible within the confines of this Review; however, brief summaries of some of the DNA repair pathways most relevant to the therapy‐induced DNA damage within the context of glioblastoma treatment will be discussed in the following section.

### 2.2 Therapy‐induced DNA lesions and associated repair mechanisms

**Fig. 2:**
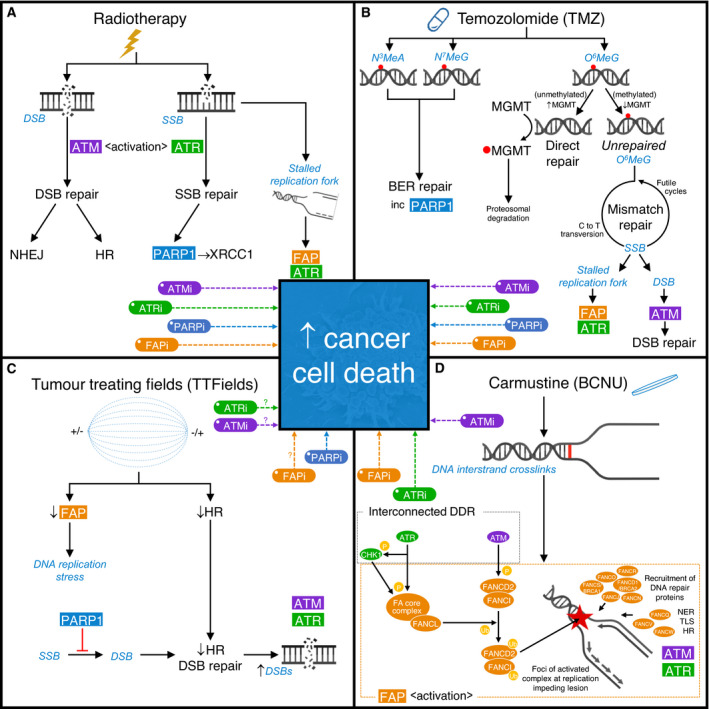
The effects of clinically approved therapies on the DNA damage response (DDR) and novel strategies to enhance efficacy of current standard‐of‐care treatments. Schematic representation of the main DNA damage lesions (in blue italic) induced by therapies approved for clinical use to treat glioblastoma and associated DDR mechanisms. For each approved treatment, putative strategies to enhance therapeutic efficacy through targeting relevant DDR mechanism(s) are indicated. (A) *Radiotherapy*: generates large amounts of DNA single‐strand breaks (SSBs) and double‐strand breaks (DSBs), which activate ATR and ATM, respectively. DSB repair is then predominantly undertaken by either nonhomologous end joining (NHEJ), which is available throughout the cell cycle but compromises fidelity, or homologous recombination (HR) DNA repair, which provides a high‐fidelity repair mechanism, but is only available during S and G2 phases of the cell cycle due to the requirement for a sister chromatid. SSB repair relies on PARP1 to detect SSBs and facilitate the recruitment of XRCC1. However, the presence of strand breaks also leads to stalling of DNA replication forks, which depend on the functions of ATR and proteins within the Fanconi anaemia pathway (FAP) for stability and replication restart. Consequently, a strong scientific rationale exists supporting inhibition of either ATM (ATMi), ATR (ATRi), PARP1 (PARPi) or the FAP (FAPi) to enhance the efficacy of radiotherapy. (B) *Temozolomide*: produces an array of methylation lesions including N3‐methyladenine (N3MeA) and N7‐methylguanine (N7MeG), which are substrates for effective removal via DNA base excision repair (BER), and O6‐methylguanine (O6MeG), which is removed directly by the enzyme MGMT in a suicide reaction. Hypermethylation of the *MGMT* gene promoter region leads to reduced MGMT expression, shifting the balance in favour of persistent O6MeG. O6MeG can act as a miscoding base during DNA replication, leading to a corresponding C‐to‐T transversion within the complementary DNA strand. If O6MeG is not successfully excised by the mismatch repair (MMR) DNA repair machinery, it endures as a perpetually miscoding base, instigating ‘futile cycles’ of MMR with consequent stalling of DNA replication forks or DSBs. (C) *Tumour‐treating fields (TTFields)*: may negatively impact FAP and HR‐mediated DNA repair processes. TTFields‐induced ‘BRCAness’ (reflecting a relative HR deficiency) provides a compelling rationale to combine this therapeutic modality with PARPi, or potentially FAPi, ATRi or even ATMi. (D) *Carmustine (BCNU) – Gliadel^®^ wafers*: provide local delivery of this bidirectional DNA alkylating agent, leading to the generation of DNA interstrand crosslinks which impede DNA replication during S phase. This leads to activation of the FAP, within which monoubiquitination of FANCD2 within the FANCD2‐I complex is a key quantifiable step. Activated FANCD2‐I coalesces as foci at sites of DNA damage and acts as a master regulator of downstream DNA repair, recruiting proteins involved in nucleotide excision repair (NER), translesion synthesis (TLS) and HR. Interplay with associated DDR mechanisms, for example ATM and ATR, leads to the phosphorylation of multiple FAP proteins (examples indicated), providing a rationale for the use of non‐FAP DDR inhibitors (e.g. ATRi or ATMi) to sensitise to crosslinking chemotherapy, and for the concept of combining multiple DDR inhibitors (including FAPi) to potentially maximise therapeutic enhancement.

Abrogation of the mechanisms that glioblastoma cells use to repair the DNA lesions induced by chemo‐ and radiotherapy may represent a key to effective multimodal treatment approaches [97] (Fig. 2). The most effective chemotherapeutic agent in current standard‐of‐care clinical use for glioblastoma is the alkylating agent temozolomide [8]. Of the array of methylation lesions induced on both nitrogen and oxygen molecules within DNA nucleotides, O6‐methlyguanine (O6MeG) is considered the most toxic lesion induced by temozolomide once metabolised from its prodrug form to MTIC [3‐methyl‐(triazen‐1‐yl)imidazole‐4‐carboxamide] [103]. O6MeG can act as a miscoding base during DNA replication, leading to a corresponding C‐to‐T transversion within the complementary DNA strand. If O6MeG is not successfully excised by the mismatch repair (MMR) DNA repair machinery [104], it endures as a perpetually miscoding base, instigating ‘futile cycles’ of MMR with consequent stalling of DNA replication forks or double‐strand breakage [104, 105]. As such, MMR capacity within GSCs harbouring genetic or epigenetic defects that affect MMR gene expression can impact on temozolomide sensitivity as well as other phenotypic traits due to the well‐established hypermutation phenotype conferred by MMR defects [106, 107, 108] (Fig. 2). Additionally, the de‐alkylating enzyme methylguanine methyltransferase (MGMT) can sequester the methyl group from O6MeG to restore the guanine residue to its original state, but this leaves MGMT irreversibly inactivated and subject to ubiquitin‐mediated proteasomal degradation [105, 109]. Consequently, high expression levels of *MGMT* can contribute to temozolomide resistance in glioblastoma [109]. On the other hand, approximately 40% of IDH‐wild‐type glioblastomas exhibit transcriptional repression of *MGMT* expression due to hypermethylation of an *MGMT*‐associated 5’ CpG island, which confers a greater benefit from temozolomide therapy and prolonged patient survival [10].

The mere notion that a DNA repair mechanism such as *MGMT* expression can profoundly influence survival for some patients provides further evidence of the critical nature of DNA repair in glioblastoma. However, it is important to note that, although both MMR activity and *MGMT* expression can impact temozolomide sensitivity of glioblastomas, longitudinal genomic profiling has revealed that such defects do not account for the majority of therapy resistance exhibited within these tumours [106]. In addition to O6MeG, the more prevalent (but less toxic) temozolomide‐induced DNA lesions N3‐methyladenine and N7‐methylguanine are primarily removed via base excision repair (BER), leaving an intermediary abasic [or apurinic/apyrimidinic (AP)] site. The nucleotide gap is subsequently filled through triggered DNA synthesis processes and strand integrity restored through ligation of the DNA ends – a process involving an AP endonuclease (APEX1), DNA polymerase (pol‐β) and DNA ligases I and III, respectively [110, 111, 112]. Additionally, nucleotide excision repair (NER) is able to remove a wide variety of structurally unrelated DNA lesions that can be generated by both chemo‐ and radiotherapy. The process can be activated by DNA helix distortions associated with structural changes to nucleotides or by stalling of RNA polymerase II due to the presence of a DNA lesion during transcript elongation, and involves the DNA unwinding before removal of a section of DNA containing the lesion followed by resynthesis using the template strand and subsequent ligation [113].

Radiotherapy exerts cellular damage by inducing a wide range of DNA lesions, but it is particularly associated with the generation of large amounts of SSBs and DSBs (Fig. 2). Poly (ADP‐ribose) polymerase‐1 (PARP1) plays a pivotal role in detection of SSBs, facilitating the colocalisation of the single‐strand break repair (SSBR) polypeptide x‐ray repair cross‐complementing protein 1 (XRCC1) [114, 115]. DNA DSBs are predominantly repaired by the nonhomologous end joining (NHEJ) and homologous recombination (HR) DNA repair pathways. The HR pathway represents a complex, high‐fidelity mechanism of DNA DSB repair. However, given its reliance on a homologous DNA sequence (duplicate DNA strand on a sister chromatid) as a template for resynthesis of removed DNA sequences around the site of DNA damage, HR‐mediated repair mechanisms are only possible during S and G2 phases of the cell cycle, when such sister chromatids are available [116, 117]. Interestingly, although TTFields therapy does not directly induce DNA breaks (unlike IR), recent work has suggested that, in addition to its effects on mitotic cells, TTFields may negatively impact on HR‐mediated DNA repair processes [16, 118], which could be important for future DDR‐targeting strategies that combine with this recently approved glioblastoma therapy. In contrast to HR, the NHEJ DNA repair pathway provides rapid DSB repair capabilities throughout the cell cycle (since a homologous sister chromatid is not required), permitting repair of a range of DNA‐end configurations (Fig. 2). However, the flexibility and rapidity of NHEJ is provided at the expense of fidelity [119, 120]. As one might expect given the cell cycle regulated nature of these pathways, both HR and NHEJ are tightly regulated by CDK activity [120, 121], a family of kinases often dysregulated in human cancers [122, 123].

Finally, the Fanconi anaemia (FA or FA/BRCA) pathway is frequently activated in response to DNA strand breaks, alkylation damage and other cytotoxic DNA lesions induced by both alkylating and DNA crosslinking chemotherapeutic agents that impede ongoing DNA replication [124, 125, 126, 127, 128]. The FA pathway is also activated during normal S‐phase progression and is regulated by both ATM and ATR kinases [124, 125, 126, 127, 128, 129, 130, 131, 132, 133, 134]. The FA pathway consists of at least 22 proteins which can be broadly categorised into three distinct functional groups (core complex, ID complex and downstream effectors) that, through sequential interaction, facilitate lesion repair and the restart of replication forks via physical removal of lesions by NER and HR‐mediated processes, as well as interactions with components of the MMR system [135].

## 3 Targeting functional interplay within the glioblastoma DDR network

### 3.1 Exploiting synthetic lethality and synthetic sensitivity strategies

As mentioned previously, there are a plethora of interconnected interactions and functional crosstalk between the various DDR, regulatory and DNA repair pathways that open up a multitude of potential therapeutic targeting strategies in heterogeneous tumours such as glioblastoma [88]. Perhaps most notable over the past 10 years or so is the concept of synthetic lethality: a concept that was originally described as a simultaneous genetic mutation in, or functional aberration of the product of, two genes which causes cell death; but in isolation, either change is survivable [136]. More recently, synthetic lethality in terms of oncology‐based therapeutic strategies has expanded to include scenarios in which specific phenotypic traits such as defective HR‐mediated DNA repair function, or aberration in the function of certain genes or pathways, result in impaired cell growth or proliferation, promoting lethal effects in the presence of additional insults such as IR or cytotoxic chemotherapy [137]. As such, the term ‘synthetic sensitivity or lethality’ (SSL) can be used to amalgamate these related concepts.

Although major advancements in the generation of RNAi and CRISPR/Cas9 libraries have undoubtedly advanced our ability to uncover potential SSL relationships at the genome scale, the most successful implementation of an SSL strategy to date in cancer treatment, the targeting of BRCA‐deficient cells with PARP1 inhibitors, was derived from hypothesis‐driven research rooted in a fundamental understanding of the specific molecular pathways concerned and relevant interplay between them, rather than broad screening approaches [138, 139]. This seminal work has led to a wave of clinical approvals for PARP1 inhibitors that are delivering survival benefits to patients with a range of cancers globally [140, 141, 142]. In the context of gliomas, tumours with IDH1/2 mutations may exhibit a ‘BRCAness’ phenotype as a consequence of inhibition of HR DNA repair processes by the enhanced levels of oncometabolites, which could explain why a subset of IDH‐mutant tumours respond well to conventional DNA damaging chemotherapies such as temozolomide as well as PARP1 and ATR inhibitors [141, 143, 144]. However, as highlighted earlier, the majority of high‐grade aggressive gliomas do not exhibit such defects in IDH genes and associated metabolism but, as discussed below, disruption to HR and associated DDR pathways may represent a credible mechanism to produce a similar phenotype in these tumours. Furthermore, challenges in efficient delivery of such compounds across the BBB are a major clinical consideration; however, at least for PARP1 inhibitors, early indications are encouraging in that therapeutically active doses can be achieved at the tumour site [145].

### 3.2 Single DDR inhibitor strategies to enhance therapeutic response

As mentioned previously, glioblastoma cells within a single tumour can demonstrate remarkable heterogeneity in the expression of DDR factors and consequently exhibit varying resistance profiles [146, 147], which likely plays a key role in treatment failure. Given the interconnected nature of the DDR pathways [88], it is perhaps not surprising that, although promising in preclinical models, targeting any single DDR pathway might not yield an effective therapeutic response clinically. The D’Andrea group were the first to demonstrate that components of the FA pathway could confer resistance to temozolomide in glioma cells [148], which was further corroborated by Kondo and colleagues [149]. Building upon these findings, we showed that the FA pathway is re‐expressed and active within high‐grade gliomas compared with low‐grade tumours as well as normal healthy tissue and that inhibition of the FA pathway in both established and primary glioma cells could confer an increased sensitivity to temozolomide [150]. These findings were recently confirmed by large‐scale CRISPR‐Cas9 screens that identified the FA and related HR pathways as key modulators of temozolomide resistance within glioma stem cells [108]. Importantly, we showed that disruption to FA pathway function was able to render glioma cells sensitive to temozolomide irrespective of *MGMT* status/expression levels [150], demonstrating a potential large scope for such an approach within the clinical setting. This is particularly important, as PARP1 inhibition (the most successful DDR‐targeting drug to date) may only confer a similar increased temozolomide sensitivity within cells that have *MGMT* promoter methylation [151], which represents ~ 40% of all patients diagnosed with glioblastoma and whose tumours are already intrinsically more sensitive to temozolomide. This is consistent with previous work by Gupta and colleagues, who demonstrated that siRNA‐mediated knockdown of either N‐methylpurine DNA glycosylase (MPG) or XRCC1 (two indispensable components of a functional BER pathway) did not confer temozolomide sensitivity in resistant glioma cell lines [152], even though PARP1 can act as a scaffold to promote BER‐mediated repair of alkylation damage [153].

Given that persistent bulky O6MeG lesions in the absence of MGMT result in elevated DNA replication stress [154, 155] and that replication fork stability and recovery is influenced by a number of DDR factors, such as PARP1, as well as the HR and FA proteins RAD51/FANCR, FANCD2, BRCA1/FANCS and BRCA2/FANCD1, the integrity of some of these interconnected DDR processes may underpin the heterogeneous preclinical success of PARP1 inhibitor‐mediated temozolomide sensitisation. At least theoretically, these data would support the use of adding an additional DDR inhibitor to maximise replication fork collapse and/or failure of resultant DNA DSB resolution, potentially resulting in more pronounced neoplastic cell death across a wider range of tumours. In this regard, it seems rational that the activity of apical DDR kinases, such as ATR and ATM, that regulate replication stress signalling and DNA break repair mechanisms might cooperate to limit the cytotoxicity of temozolomide in cancer cells. Interestingly, activation of both ATR and ATM following temozolomide chemotherapy occurs in an MMR‐dependent manner [156], which likely reflects the nature of the cytotoxicity associated with O6meG compared with N7‐meG and N3‐meA lesions, which do not activate MMR. Previous work by Eich and colleagues showed that siRNA‐mediated knockdown of either ATR or ATM sensitised an MGMT‐negative glioma cell line to temozolomide [157], with similar findings for ATM also reported by Nadkarni *et al*. [158]. Interestingly, the degree of sensitisation conferred by ATR knockdown in the Eich study was more than double that observed following *ATM* siRNA, indicating that impaired resolution of DNA replication stress generated from O6MeG lesions may be a key driver of sensitisation in this context, especially given that simultaneous ATR and ATM knockdown did not provide any additional sensitisation relative to ATR alone and that the enhanced temozolomide sensitisation could be rescued by ectopic expression of MGMT [157]. These findings were also recently corroborated by Jackson *et al*., who established that MGMT‐deficient glioma cells are profoundly susceptible to temozolomide sensitisation using small‐molecule ATR inhibitors both *in vitro* and *in vivo* [159].

### 3.3 The FA pathway as a foundation for future DDR‐centric combinatorial strategies

In addition to potential compounding genetic and/or epigenetic alterations within glioblastomas that might affect DDR‐targeting strategies, and the interplay within the BER, MMR, MGMT, FA and HR pathways (highlighted above), interactions between the FA pathway and other DDR elements have been characterised that are also important to consider with regard to potential FA‐based combination targeting strategies within glioblastomas [160]. For example, as mentioned previously, both ATM and ATR phosphorylate several proteins within the various FA subcomplexes [161, 162, 163] and components of the FA pathway have also been shown to promote activation of ATR and supress potentially deleterious repair of DNA ICLs by NHEJ [164, 165, 166]. Additionally, as the FA pathway promotes HR‐mediated DNA repair processes around stalled/collapsed replication forks [167], HR‐deficient cells have been shown to be sensitive to disruption of FA pathway function [168, 169]. Given this functional interplay, it has been postulated that FA dysfunctional tumours, or targeting of the FA pathway, may render them sensitive to PARP1 inhibitors [170, 171, 172], as well as a multitude of other drug targeting strategies within the FA and related DDR pathways [170]. Indeed, we have recently generated data in clinically relevant GSC 3D cell culture model systems highlighting profound radio‐ and chemosensitisation through the combined targeting of the FA pathway in combination with ATM, ATR or PARP1 inhibitors (Rominiyi *et al*, manuscript under preparation).

Unfortunately, there have been no reported rationally designed inhibitors of the FA pathway to date; however, recent structural insights into key regulatory components of the FA pathway together with current efforts by several groups to identify FA pathway inhibitors (including our own group) [173, 174, 175, 176, 177, 178] should hopefully lead to the emergence and further development of such compounds in the near future that could be of great clinical significance for glioblastoma treatment regimes. Apart from exhibiting high potency and specify, such compounds would also need to demonstrate high biological efficacy within the correct brain regions or be adaptable to novel delivery mechanisms in order to maximise their effectiveness in any such therapeutic regimes [3]. Encouragingly, tumour margin penetration within a biologically active drug concentration range has been recently reported for the PARP1 inhibitor olaparib (Lynparza) in combination with temozolomide chemotherapy as part of the OPARATIC trial [145], and it will be interesting to see the results from other DDR inhibitor trials for glioblastoma when they are reported (Table 1).

**Table 1:** DNA damage response inhibitor trials in high‐grade glioma. AEs^(G3‐4)^, grade 3–4 adverse events; AEs, adverse events; and WBRT, whole brain radiotherapy; CR, complete response; DIPG, diffuse intrinsic pontine glioma; DLTs, dose‐limiting toxicities; EFS, event‐free survival; F/U, follow‐up; IMRT, intensity‐modulated radiation therapy; IR, radiotherapy; MTD, maximum tolerated dose; nGBM, newly diagnosed GBM; NIRA, niraparib; OLAP, olaparib; ORR, overall response rate (proportion of patients with a PR or CR); OS, median overall survival; PAMI, pamiparib; PFS, median progression‐free survival; PR, partial response; QoL, quality of life; rGBM, recurrent GBM; rHGG, recurrent high‐grade glioma; RP2D, recommended phase II dose – highest dose with acceptable toxicity (producing a rate of around 20% DLTs); Rx, treatment; SAEs, severe adverse events; SD, stable disease; SoC, standard‐of‐care; TALA, talazoparib; TMZ, temozolomide; TTF, tumour‐treating fields; VELI, veliparib.

Trial (reference) & indication	Design & n (rec dates)	Treatment(s)	1° Endpoint	2° Endpoint(s)	Results/remarks & conclusions
*Summary of key PARP inhibitor in high‐grade glioma clinical trials – completed and ongoing*					
Phase I studies					
*NCT00770471* [228] nGBM Veliparib (ABT‐888), radiation therapy, and temozolomide in treating patients with newly diagnosed glioblastoma multiforme	Phase I Single arm 24 patients (2009–2012)	VELI + IR + TMZ	Phase I: VELI MTD Phase II: OS (with VELI MTD)	A) Safety/toxicity B) Pharmacokinetics	Following initial safety groups and planned dosing steps, 3/6 pts (50%) had DLTs (2 thrombocytopenia, 1 neutropenia) with 10mg BD VELI+R+TMZ → accrual discontinued VELI at this dose with standard dosing regimen of IR+TMZ deemed not tolerable Further development of appropriate dosing regimen needed
*NCT01390571* [229, 230] rGBM Olaparib and temozolomide in treating patients with relapsed glioblastoma (OPARATIC)	Phase 0/I Single arm 48 patients (2011–2017)	Stage I: OLAP for 3/7 prior to surgery then usual Rx Stage II: Escalating OLAP 3/7 prior to surgery then OLAP + TMZ post‐op	Phase 0: Tumour penetration via BBB/BTB Phase I: Safety	A) BBB disruption/permeability B) Preliminary antitumour activity of OLAP + TMZ	OLAP detected in 73/74 tumour specimens from 27 pts, mean conc. 588nM (range = 97–1374 nm). Mean tumour margin : core ratio = 1.2 (0.2–3.9) Mean tumour : plasma ratio = 0.25 (0.01–0.9) 24/35 pts (67%) AEs^(G3‐4)^ 45% PFS at 6 m F/U OLAP penetrates tumour core/margins and is safe with extended low‐dose TMZ
*NCT01294735* [230] rGBM/rMelanoma/solid cancers Niraparib (MK‐4827) given with temozolomide in participants with advanced cancer	Phase I Single arm 19 patients (2011–2012)	NIRA + TMZ	No. of DLTs	A) ORR within 30 days of last dose & 2m intervals B) PFS	MTD & RP2D = 40 mg OD NIRA with 150 mg·m^−2^ TMZ. 2/10 pts (20%) had Grade 4 thrombocytopenia at this dose 1 PR (glioblastoma) & 2 SD out of 16 evaluable pts NIRA tolerable in combination with TMZ
nGBM [231] Two parallel phase I studies of olaparib and radiotherapy or olaparib and radiotherapy plus temozolomide in patients with newly diagnosed glioblastoma, with treatment stratified by MGMT status (PARADIGM‐2)	Phase I Parallel Estimated patients: 25–40 methylated; 19–28 unmethylated (2016–2021)	Methylated: OLAP + IR + TMZ Unmethylated: OLAP + IR	Safety/toxicity (MTD & optimum scheduling)	A) Define DLTs (+/− TMZ)	MGMT methylated dosing schedule = OLAP (dose escalation) with IR and concomitant TMZ, then 4 weeks OLAP with maintenance TMZ started *after* completing OLAP Trial ongoing – recruitment ends May 2021
Phase I/II studies					
*NCT01026493* [232] rGBM A randomized phase I/II study of veliparib (ABT‐888) in combination with temozolomide in recurrent (temozolomide resistant) glioblastoma (RTOG0929)	Phase I/II Randomised 225 patients: 151 BEV naïve (BEV‐N); 74 BEV refractory (BEV‐R) (2009–2017)	Arm 1: VELI + TMZ 75 mg·m^−2^ (both 21/28 day cycle) Arm 2: VELI + TMZ 150 mg·m^−2^ (both 5/28 day cycle)	Phase I: MTD. Phase II: PFS at 6m	A) ORR B) OS	Myelosuppression AE^(G3‐4)^ in 20% of pts PFS at 6 m = 17.0% (BEV‐N) & 4.4% (BEV‐R) – median PFS ~ 2 m (95% CI, 1.9–2.1 m) in both groups Median OS = 10.3 m (8.4–12.0 m, BEV‐N) & 4.7 m (3.5–5.6 m, BEV‐R) Concluded addition of VELI ‘did not significantly improve PFS at 6m’ relative to historic controls Note: MGMT status was not included or analysed
*NCT01514201* [214, 233] DIPG Veliparib, radiation therapy, and temozolomide in treating younger patients with newly diagnosed diffuse pontine gliomas: a paediatric brain tumor consortium study	Phase I/II Single arm 65 patients (2012–2018)	VELI + IR + TMZ	Phase I: RP2D/DLTs. Phase II: OS	A) PFS B) Pseudoprogression C) Pharmacokinetics	VELI RP2D was 65 mg·m^−2^ BD Day 4 average VELI (65 mg·m^−2^) plasma *C*_max_ = 3 μm VELI DLTs inc: intratumoural haemorrhage (1 pt, Grade 2); rash (2 pts, Grade 3); neurological (1 pt, Grade 3) Additional intrapatient TMZ dose escalation could not be tolerated OS at 1 and 2 years = 37.2% and 5.3% respectively Accrual stopped early due to futility at interim analysis
*NCT02116777* [234] Solid & haematological cancers Talazoparib and temozolomide in treating younger patients with refractory or recurrent malignancies	Phase I/II Single arm 40 patients (2014–2018)	TALA + TMZ	Phase I: MTD/RP2D; Safety/toxicity; Pharmacokinetics Phase II: ORR (Ewing/PNET)	A) ORR all solid tumours (RECIST)	RP2D = TALA 600 μg·m^−2^ BD on day 1 then OD days 2–6/28 with TMZ 30 mg·m^−2^ day 2–6/28 cycle Majority of patients had Ewing sarcoma (EWS), but one patient with a malignant glioma experienced a PR During Phase II, no response observed out of 10 EWS pts No efficacy in EWS but may warrant further study in CNS tumours
*NCT03150862* [235] nGBM/rGBM Pamiparib (BGB‐290) with radiation and/or temozolomide (TMZ) in newly diagnosed or recurrent glioblastoma	Phase Ib/II Parallel Estimated patients: 116 (2017–2021)	nGBM (unmethylated) Arm 1: PAMI + IR Arm 2: PAMI + IR + TMZ rGBM (un‐ & methylated) Arm 3: PAMI + TMZ	Phase I: Safety/toxicity Phase II: Disease response/control	A) Pharmacokinetics B) PFS C) OS D) ORR	RP2D for Arm 1 = PAMI 60 mg BD for 6 weeks alongside IR RP2D for Arm 3 = PAMI 60 mg BD day 1–28 + TMZ 60 mg·m^−2^ 7/28 day cycle Well tolerated – no Grade 4/5 toxicities; Grade 3 – Arm 1 ‐nausea (2%), Arm 2 – decreased WBC count (11%). Arm 3 none PAMI + IR + TMZ well tolerated – trial ongoing – recruitment ends October 2021, final results awaited
*NCT03212742* [236] Unresectable HGG Study of concomitant radiotherapy with olaparib and temozolomide in unresectable high‐grade gliomas patients (OLA‐TMZ‐RTE‐01)	Phase I/IIa Sequential Estimated patients: 79 (2017–2022)	OLAP + IR + TMZ	Phase I: RP2D for both IR‐period and maintenance period. Phase II: OS	A) PFS B) ORR C) Neurocognitive function D) Morphological and functional MRI findings	Dosing schedule = OLAP (IR‐period dose) with IR and concomitant TMZ, then 4 weeks OLAP at same dose → then maintenance TMZ started alongside daily OLAP (maintenance dose) Trial ongoing – expected completion June 2022
Phase II studies					
*NCT02974621* rGBM Cediranib maleate and olaparib compared to bevacizumab in treating patients with recurrent glioblastoma	Phase II Randomised Estimated patients: 70 (2017–2020)	Arm 1: OLAP + cediranib Arm 2: BEV	PFS	A) OS B) Safety/toxicity C) Circulating biomarkers (inc DDR and cytokines)	Dosing schedule = OLAP BD on day 1–28/28 cycle with cediranib OD on day 1–28/28 cycle Trial ongoing – recruitment completed May 2020, results awaited (estimated study completion date May 2021)
*NCT03233204* Solid tumours with DDR defects Phase 2 subprotocol of olaparib in patients with tumors harbouring defects in DNA damage repair genes (NCI‐COG Paediatric MATCH (Molecular Analysis for Therapy Choice))	Phase II Single group assignment Estimated patients: 49 (2017–2024)	OLAP only	ORR	A) PFS B) Safety/toxicity C) Pharmacokinetics	Patient subprotocol assignment from within the overall paediatric MATCH study [237] – based on actionable mutations Eligible actionable mutations not defined at trial registration Dosing schedule = OLAP BD on day 1–28/28 cycle Changes in tumour genomic profile monitored using ctDNA Trial ongoing – recruitment ends September 2024
*NCT03212274* rGlioma (WHO Grade II‐IV) / cholangiocarcinoma / solid tumours with IDH1/2 mutation Olaparib in treating patients with advanced glioma, cholangiocarcinoma, or solid tumours with IDH1 or IDH2 mutations	Phase II Single arm Estimated patients: 145 (2018–2021)	OLAP only	ORR (3 cohorts) A) Glioma B) Cholangio C) Other solid tumours	A) PFS B) OS C) Safety/toxicity D) Exploratory objectives inc correlation between baseline 2HG and response	Dosing schedule = OLAP BD on day 1–28/28 cycle Builds on preclinical studies demonstrating ‘BRCAness’ with IDH1/2 mutation and elevated 2HG [238] Trial ongoing – recruitment ends July 2021, results awaited
*NCT03581292* nHGG (H3K27M^−^ BRAFV600^−^) Veliparib (ABT‐888), radiation therapy, and temozolomide in treating patients with newly diagnosed malignant glioma without H3 K27M or BRAFV600 mutations	Phase II Single arm Estimated patients: 115 Age 3–25 (2018–2024)	VELI + IR + TMZ	PFS	A) ORR B) OS	Dosing schedule = daily VELI BD during chemoradiotherapy phase then 4 weeks after completion → daily VELI BD + maintenance TMZ on days 1–5/28 cycle Incorporates longitudinal assessment of ctDNA Exploratory objectives inc: relationship between BRCA1/2 alternations and features of HRD (inc. large‐scale translocations, mutational signature 3); penetrance of HRD genes inc. HR genes, FA genes, ATM, CHK2, and MMR genes Trial ongoing – recruitment ends October 2024
*NCT04221503* rGBM Evaluating the efficacy and safety of niraparib and tumor‐treating fields in recurrent glioblastoma (Niraparib/TTFields) [240]	Phase II Parallel Estimated patients: 30 (2019–2025)	All patients receive NIRA + TTFields Cohort 1: surgical resection indicated Cohort 2: resection not indicated	Disease control (CR/PR or SD)	A) Safety/toxicity B) ORR C) PFS D) OS	Cohort 1: initiate and continue TTFields for 5–7 days prior to starting NIRA Cohort 2: receive TTFields for 5–7 before planned resection, then postoperative therapy as above Builds on preclinical studies demonstrating ‘BRCAness’ induced by TTFields [118, 239] Trial ongoing – expected completion September 2021
*NCT03561870* rHGG (IDH^mut^) Olaparib in Recurrent IDH‐mutant High Grade Gliomas (OLAGLI)	Phase II Single arm Estimated patients: 35 (2020–2021)	OLAP only	PFS	n/a	Dosing schedule = OLAP 300 mg BD on days 1–28/28 cycle Based on preclinical studies demonstrating ‘BRCAness’ with IDH1/2 mutation and elevated 2HG [238] Trial ongoing – expected completion September 2021
*NCT03991832* IDH^mut^ solid tumours Olaparib and durvalumab in patients with IDH‐mutated solid tumors (MEDI 4736)	Phase II Parallel Estimated patients: 78 (2020–2022)	All pts receive OLAP + durvalumab Cohort 1: Glioma Cohort 2: Cholangio Cohort 3: Other solid tumours	ORR	A) PFS B) OS C) Safety/toxicity	Dosing schedule = OLAP BD on days 1–28/28 cycle + durvalumab (anti‐PD‐L1 therapy) on day 1/28 cycle Based on preclinical studies demonstrating ‘BRCAness’ with IDH1/2 mutation and elevated 2HG [238] Trial ongoing – recruitment ends September 2022, expected completion September 2023
Phase II/III studies					
*NCT02152982* nGBM (MGMT promoter hypermethylated) Temozolomide with or without veliparib in treating patients with newly diagnosed glioblastoma multiforme	Phase II/III RCT Randomised 447 patients (2014–2021)	After SoC IR and concomitant TMZ: Arm 1: VELI + TMZ Arm 2: PLACEBO + TMZ	OS	A) ‘Interaction’ with TTFields (for pts receiving this) B) PFS C) ORR D) Safety/toxicity E) QoL	Patients permitted to receive TTFields alongside trial therapies. No other additional therapies permitted Studies will also assess whether genetic/epigenetic alternations to DDR genes influence outcomes Trial ongoing – recruitment completed November 2020, results awaited
*Summary of key ATM inhibitor in high‐grade glioma clinical trials – ongoing*					
Phase I studies					
*NCT03423628* [241, 242] nGBM/rGBM/brain metastases Safety and tolerability of AZD1390 given with radiation therapy in patients with brain cancer	Phase I Parallel Estimated patients: 132 (2018–2023)	AZD1390 + SoC IR: nGBM: IMRT 60 Gy over 6 weeks rGBM: IMRT 35 Gy over 2 weeks Mets: WBRT 30 Gy over 2 weeks	Safety/toxicity	A) EFS B) ORR C) Pharmacokinetics	Dosing schedule = AZD1390 administered in 3 ‘cycles’ – (1) 1 dose prior to starting IR; (2) intermittent if continuous administration during IR; (3) 2‐week adjuvant ATMi after IR Based on preclinical studies demonstrating BBB penetration and improved survival with AZD1390 in mouse models [243] Trial ongoing – expected completion February 2023
*Summary of key DNA‐PK inhibitor in high‐grade glioma clinical trials – ongoing*					
Phase II studies					
*NCT02977780* nGBM INdividualized Screening Trial of Innovative Glioblastoma Therapy (INSIGhT)	Phase II Parallel RCT Estimated patients: 250 (2017–2021)	After SoC IR and concomitant TMZ: Arm 1: TMZ Arm 2: Neratinib + TMZ Arm 3: CC115 + TMZ Arm 4: Abemaciclib + TMZ	OS	A) Safety/toxicity B) PFS C) Biomarkers & survival associations	Compares SoC therapy with 3 novel regimens each adding an additional drug: neratinib (tyrosine kinase inhibitor); CC115 (DNA‐PK inhibitor); abemaciclib (cyclin‐dependent kinase 4 and 6 inhibitor) Details on CC115 dosing schedule not available Trial ongoing – expected completion December 2022
*Summary of key WEE1 inhibitor in high‐grade glioma clinical trials – ongoing*					
Phase I studies					
*NCT02207010* [225] rGBM A phase 0 study of AZD1775 in recurrent GBM patients	Phase 0/I Single arm 20 patients (2015–2019)	Single dose of AZD1775 (100mg, 200mg or 400mg) prior to surgery	A) Plasma concentration B) Intratumoural concentration	Tissue biomarker analysis	Mean peak total AZD1775 plasma concentration over 100 ng·mL^−1^ with single 200 mg or 400 mg dose Mean unbound AZD1775 tumour concentration of 85 ng·g^−1^ at 2–24 h exceeding the *in vitro* IC_50_ (40 ng·mL^−1^) for WEE1 inhibition Confirmation of target effects including elevated γH2AX, pH3 and cleaved caspase‐3
*NCT01849146* [244] nGBM/rGBM Adavosertib (AZD1775), radiation therapy, and temozolomide in treating patients with newly diagnosed or recurrent glioblastoma	Phase I Nonrandomised Estimated patients: 114 (2013–2021)	Arm 1: AZD1775 during initial IR + TMZ and maintenance TMZ Arm 2: AZD1775 during maintenance TMZ	A) MTD B) Safety/toxicity	A) OS B) PFS	Preliminary data suggests AZD1775 in combination with initial IR + TMZ at 150 mg QDS and 425 mg QDS alongside maintenance TMZ for 5 days in each 28 day cycle had acceptable toxicity Trial recruitment completed – estimated study completion December 2021

### 3.4 Expanding the preclinical evidence base for parallel targeting of the DDR

As highlighted previously, there has been intense research into the development of small‐molecule inhibitors of DDR proteins [82]; to date, however, only a handful of published preclinical studies have examined combined inhibition of multiple DDR elements simultaneously in glioblastoma. Given that ATM and PARP1 inhibition each individually possess some utility in sensitising GSCs to radiotherapy [179, 180], Ahmed *et al*. [181] investigated parallel inhibition of the DDR targets ATM, ATR, CHK1 and PARP1 in primary patient‐derived glioblastoma cell lines. Firstly, in agreement with previous findings by Bao *et al*. [48], this study demonstrated that these DDR factors were upregulated in the inherently treatment‐resistant subpopulation of GSCs compared to bulk populations. This further confirms the importance of targeting multiple DDR pathways and the potential for functional redundancy within the DDR to contribute to the treatment‐refractory nature of glioblastoma. Secondly, the study by Ahmed *et al*. demonstrated that combined inhibition of PARP1 and ATR resulted in a profound radiosensitisation of GSCs, with effects greater than any single inhibitor used in isolation [181].

The rationale for a combination DDR inhibitory strategy in glioblastoma is also supported by the work of Signore and colleagues, who performed a simultaneous multipathway approach with subsequent reverse‐phase protein microarrays and kinase inhibitor library screening to identify dual inhibition of CHK1 and PDK1, resulted in profound retardation of GSC growth in both *in vitro* and *in vivo* (subcutaneous and intracranial mouse) models [182]. Although this study is highly informative, the lack of target specificity with use of a drug such as UCN‐01 severely limits potential clinical utility due to the high likelihood of dose‐limiting toxicities mediated by known off‐target effects associated with this compound. Nevertheless, this work provides further preclinical proof‐of‐concept data in support of such combinatorial approaches within heterogeneous glioblastoma tumour populations. More recently, Rasmussen *et al*. [183] demonstrated that reduced DDR capacity through PARP1 inhibition (olaparib) in conjunction with epigenetic‐downregulation‐induced oxidative stress through histone deacetylase (HDAC) inhibition (vorinostat) led to reduced glioblastoma cell survival, induced apoptosis and impaired cell cycle progression.

**Fig. 3:**
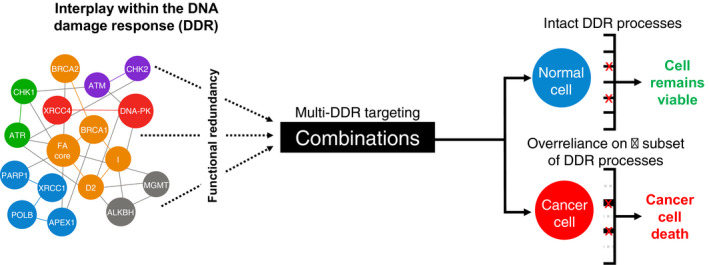
An approach for cancer‐selective killing through multimodality targeting of interconnected DNA damage response (DDR) pathways. A schematic representation of simultaneous targeting of multiple interconnected DDR processes to achieve cancer‐selective killing. *Left* – a simplified network schematic of key DDR proteins illustrating the complexity of intra‐ and interpathway protein–protein interactions within the global DDR. This complexity provides a degree of functional redundancy in DDR processes, which is likely to afford therapeutic resistance to current DNA damaging therapies. *Right* – due to the loss of functionality within some DDR pathways during carcinogenesis, cancerous cells often demonstrate overreliance on a reduced subset of DDR processes for cell survival. Where inhibition of a single DDR pathway may not be sufficient to provide synthetic lethality or substantial cancer cell killing, targeting multiple DDR processes simultaneously may overwhelm the remaining functional DDR leading to exquisitely potent cancer cell killing. However, by virtue of their complete repertoire of fully functional DDR processes, normal cells might continue to avoid significant toxicity associated with multi‐DDR‐targeting strategies (e.g. PARPi in noncancerous breast tissue that exhibits normal BRCA1/2 expression/function).

As demonstrated through these examples, the principle of multimodal targeting within the DDR network based on an in‐depth mechanistic understanding of functional interplay and regulatory mechanisms offers a potentially powerful approach to combat biological complexity and functional redundancy within the DDR, as well as intratumour heterogeneity within glioblastoma tumour subpopulations (Fig. 3). Indeed, preclinical research investigating such dual DDR inhibition approaches outside of glioblastoma research is supportive of this concept, for example CHK1 and PARP1 inhibition in pancreatic cancer [184], CHK2 and PARP1 inhibition in lymphoma [185], CHK1 and PARP1 inhibition in breast cancer [186], ATR and PARP1 inhibition in breast and ovarian cancer [187], and ATM and PARP1 inhibition in lung cancer [188].

### 3.5 Indirect DDR‐targeting strategies in glioblastoma

In addition to direct combination targeting of DDR network factors, a comparable approach can be to target other signalling pathways that impact on DDR activity and/or capacity. For example, Gomez‐Roman *et al*. [64] recently demonstrated in 3D GSC models that disruption to functional VEGF and AKT signalling pathways impacted the balance between NHEJ and HR DNA DSB break repair activities to increase radiation sensitivity. This is particularly interesting given that targeted therapies against VEGF (such as bevacizumab) have generally failed to improve patient overall survival in large clinical trials [51].

In a similar manner to identifying nonclassical DDR pathway targeting strategies, we recently identified ERK5/MAPK5 through a kinome‐wide RNAi screen as a novel temozolomide resistance factor, with abrogation of ERK5 activity in glioma cells leading to defective DNA repair capacity, likely through inappropriate NHEJ activity prior to mitosis [189]. Interestingly, ERK5 has recently been identified as a key factor in promoting cell growth and cell survival in the aggressive diffuse intrinsic pontine gliomas [190], supporting recent evidence around ERK5 as an emerging novel oncology drug target [191, 192, 193]. As such, we are currently further assessing the potential of ERK5 targeting in glioblastomas as part of various combinatorial approaches together with current standard‐of‐care therapy.

On the topic on non‐DDR signalling kinases that impact the DDR, Riess *et al*. [194] recently assessed targeting of the CDK family of signalling kinases given their common dysregulation in glioblastoma and the recent advancement of a new generation of clinically approved compounds. Using a CDK‐based monotherapy approach in various 3D glioblastoma preclinical models, they showed that CDK inhibitors could negatively affect tumour growth, but also that some CDK inhibitors were able to effectively combine with DNA damaging regimes such as radiation and temozolomide treatments. However, they also showed that not all tested CDK inhibitors behaved in the same way, with some conferring antagonistic properties when combined with temozolomide, potentially through differential effects on global gene expression patterns [194]. This study further highlights the need to understand the intricate functional interplay and regulatory mechanisms within the DDR as part of preclinical studies to help maximise the therapeutic potential of such new combinatorial regimes within the clinic.

In addition to CDK dysregulation, another common feature of cancer cells is a heightened level of replication stress due to the activation of oncogenes [92, 195]. Oncogene‐induced replication stress has been shown to be present in GSCs [49] and is capable of triggering the DDR during early tumorigenesis [196]. Recent work from Ning *et al*. [197] revealed that heightened MYC activity in GSCs can lead to suppression of ATR‐mediated replication stress signalling through transcriptional repression of CDK18. This is consistent with recent findings from our laboratory that identified CDK18 as a novel component of the ATR‐mediated replication stress signalling module that promotes cellular resistance to a variety of replication stress‐inducing chemotherapeutic agents [198, 199]. In keeping with the aforementioned interplay between ATR signalling and PARP1 activity (see above), Ning *et al*. [197] further showed that GSCs with reduced CDK18 expression and subsequent retarded ATR activity, as a consequence of oncogenic MYC action, were rendered sensitive to PARP1 inhibitors. In keeping with a heightened S‐phase fraction within a subpopulation of GSCs, a recent study by Zhou *et al*. [200] showed that purine metabolism was increased in aggressive/high‐grade tumours and represents a potential target to improve the effectiveness of radiotherapy regimes. This is especially compelling given that, as highlighted within this study, there are currently FDA‐approved inhibitors of GTP synthesis, although obviously the efficient delivery of therapeutic doses within the brain will be key to the success of such strategies.

Finally, another common feature of cancer cells, particularly solid tumours, is an imbalance between oxygen supply and demand from active aerobic metabolism, causing regional hypoxia defined as regions of reduced oxygen concentration. This presents both challenges in terms of cell death mechanisms, which are less effective in the context of hypoxia, such as those elicited by radiotherapy, but also potentially exploitable therapeutic opportunities given the effects hypoxia has on several DDR factors [201, 202, 203, 204]. Recent discoveries outside of glioblastoma have revealed key molecular and functional links between the DDR, replication stress signalling and the cGAS‐STING immune pathways [205, 206], and that targeting of replication stress signalling may synergise with immuno‐oncology (IO) therapies [207, 208]. However, the propensity of glioblastoma to escape immunosurveillance, potentially poor receptor expression and anatomical considerations have so far limited progress in the development of effective immunotherapies for glioblastoma compared to other cancers [209, 210, 211, 212]. However, strategies to circumvent such immunosurveillance escape in gliomas have recently been reported [213] and, as more mechanistic understanding around how these pathways interact becomes available, further therapeutic opportunities for gliomas will hopefully be developed. Together, these studies raise the possibility that oncogene‐induced replication stress within residual GSC populations following surgical resection may be targeted with agents that exploit such defective ATR signalling, for example. However, as is unfortunately all too common in glioblastoma research, promising preclinical studies do not necessarily translate into clinical benefit for patients [3, 214] so expectations in this regard need to be measured.

## 4 Conclusions and future perspectives

As highlighted in this Review, there is a critical need for new and more effective treatment strategies to combat the long‐standing dismal survival rates experienced by patients diagnosed with high‐grade brain tumours such as glioblastoma. Through the combined acquisition of fundamental biological insights into DDR mechanisms and interplay within this network and associated pathways, together with continued progress in imaging, surgical technologies and radiochemotherapy delivery within the clinic, it is expected that targeting of the DDR has a potential to tackle the historic lack of treatment options for these tumours. It will be particularly interesting to see the results of DDR inhibitor clinical trials currently in various phases around the world (Table 1), as this will give an indication of how successful such approaches may or may not be [145, 215]. There is, of course, the risk, given the extensive intratumoural heterogeneity of glioblastoma and inherently treatment‐resistant GSCs within these tumours, that any single targeted therapy may in fact be ‘too targeted’, leading to inevitable resurgence of resistant subclones and tumour repopulation. In a similar manner to other difficult‐to‐treat diseases such as HIV and multidrug‐resistant tuberculosis [216], novel drug combinations may be required to overcome the extensive genetic heterogeneity and resistance mechanisms observed in glioblastoma. Consequently, as highlighted within this Review, targeting multiple DDR constituents in parallel has the potential to provide new effective treatment paradigms that might help prevent disease progression by counteracting complex and overarching phenotypic delinquency within the DDR of cancerous cells (Fig. 3).

In addition to the aforementioned needed improvement in preclinical models that better reflect postsurgical residual disease, an important factor alongside the development of such multimodal strategies will be further improvements in the efficient delivery of therapeutically active doses of drugs beyond the BBB, which remains a significant challenge in glioblastoma therapy [3]. Potential innovations on the horizon include the use of MRI‐directed magnetic nanoparticles [217, 218], surgical delivery of *in situ* gelling agents [219, 220] and enhanced intrathecal/cerebrospinal fluid delivery using novel viral vectors, antibody ligands or exosomes [221, 222, 223]. Such approaches, in addition to traditional oral or intravenous delivery approaches, coupled with novel ways to disrupt the BBB, such as ultrasound [224, 225] or TTFields [226]‐based approaches, will hopefully provide the best chance for DDR‐targeting approaches to provide much‐needed clinical benefit to patients and families faced with the devastating diagnosis of a high‐grade glioma.

## Conflict of interest

OR and SJC have received research funding from the funding bodies acknowledged below and are recipients of an Inovitro™ TTFields preclinical research system (on loan from Novocure) and take part in the annual Inovitro™ Users Meeting hosted by Novocure.

## Author contributions

OR and SJC conceived and wrote the manuscript following an invitation from Prof. Kevin Ryan (co‐EIC).
